# GaitRec, a large-scale ground reaction force dataset of healthy and impaired gait

**DOI:** 10.1038/s41597-020-0481-z

**Published:** 2020-05-12

**Authors:** Brian Horsak, Djordje Slijepcevic, Anna-Maria Raberger, Caterine Schwab, Marianne Worisch, Matthias Zeppelzauer

**Affiliations:** 1 St. Pölten University of Applied Sciences, Institute of Health Sciences, St. Pölten, Austria; 2 St. Pölten University of Applied Sciences, Institute of Creative Media Technologies, St. Pölten, Austria; 3 Rehabilitation Center Weißer Hof, Austrian Workers’ Compensation Board (AUVA), Klosterneuburg, Austria

**Keywords:** Biomedical engineering, Machine learning, Outcomes research

## Abstract

The quantification of ground reaction forces (GRF) is a standard tool for clinicians to quantify and analyze human locomotion. Such recordings produce a vast amount of complex data and variables which are difficult to comprehend. This makes data interpretation challenging. Machine learning approaches seem to be promising tools to support clinicians in identifying and categorizing specific gait patterns. However, the quality of such approaches strongly depends on the amount of available annotated data to train the underlying models. Therefore, we present GaitRec, a comprehensive and completely annotated large-scale dataset containing bi-lateral GRF walking trials of 2,084 patients with various musculoskeletal impairments and data from 211 healthy controls. The dataset comprises data of patients after joint replacement, fractures, ligament ruptures, and related disorders at the hip, knee, ankle or calcaneus during their entire stay(s) at a rehabilitation center. The data sum up to a total of 75,732 bi-lateral walking trials and enable researchers to classify gait patterns at a large-scale as well as to analyze the entire recovery process of patients.

## Abstract (summary)

**Table:** 

Measurement(s)	gait measurement • phenotypic annotation
Technology Type(s)	force sensor • visual observation method
Factor Type(s)	experimental condition • musculoskeletal impairment • age • sex • shod condition • walking speed
Sample Characteristic - Organism	Homo sapiens
Sample Characteristic - Environment	laboratory environment

Machine-accessible metadata file describing the reported data: 10.6084/m9.figshare.12162300

## Background & Summary

The quantification of ground reaction forces (GRF) is a standard tool for clinicians to objectively measure human locomotion and to describe and analyze a patient’s gait performance in detail. The primary aim of instrumented gait analysis, regardless of which technology used, is to identify impairments that affect a patient’s gait pattern and to describe those quantitatively^1^. Recordings obtained during clinical gait analyses produce a vast amount of data which are difficult to comprehend and analyze due to their high-dimensionality, temporal dependencies, strong variability, non-linear relationships and correlations within the data^2^. This makes data interpretation challenging and requires an experienced clinician to draw valid conclusions. Therefore, there is a constantly growing interest in applying machine learning techniques to clinical gait analysis data for the purpose of pattern identification and automated classification. Such systems might bear potential to assist clinicians in identifying and categorizing specific gait patterns into clinically relevant categories^2,3^. Machine learning methods employed in this context comprise, but are not limited to, neural networks^4–6^, support vector machines^7–9^, nearest neighbor classifiers^10,11^, and different clustering approaches^12^.

Our research group is collaborating with a local Austrian rehabilitation center of the Austrian Workers’ Compensation Board (AUVA). The AUVA is the social insurance for occupational risks for more than 3.3 million employees and 1.4 million pupils and students in Austria. They have been using GRF assessments during walking to diagnose, plan and evaluate therapy outcomes for more than two decades. Our main research goal within this collaboration was to develop automatic classification algorithms which support clinicians during data inspection and interpretation. To this end, we have developed a machine learning framework for gait classification and have performed comprehensive experiments^13–16^. One conclusion of our experiments is that the performance of automatic classification methods strongly depends on the amount of available training data. One reason for this is that state-of-the-art classifiers such as deep neural networks^17^ are extremely data hungry and require large-scale data to learn meaningful and generalizable patterns from the data. The training process, however, requires each walking-trial in the dataset to be annotated and categorized exactly. Even though there are datasets available relevant to instrumented gait analysis, e.g.^18^, the availability of completely annotated large-scale datasets is very scarce. Our collaboration with the AUVA and their gait laboratory gave us the unique opportunity to process and manually annotate thousands of walking GRF trials from several years of clinical practice. These data have been used in our previous research and show a large potential for further research in gait analysis (see section usageNotes) to achieve the long-term goal to put assistive machine learning techniques into clinical gait analysis practice. For this purpose, we make these data available to the public as the GaitRec dataset.

## Methods

### Data recording & testing protocol

The presented dataset is part of an existing clinical gait database maintained by a local Austrian rehabilitation center, which offers care to patients across entire Austria. Prior to the experiments involved and the publication of the dataset, approval was obtained from the local Ethics Committee of Lower Austria (GS1-EK-4/299-2014). Data were recorded during clinical practice between 2007 and 2018. Bi-lateral GRF were recorded by asking patients and healthy controls to walk unassisted and without a walking aid at self-selected walking speed on an approximately 10 m walkway with two centrally embedded force plates (Kistler, Type 9281B12, Winterthur, CH). The force plates were placed in a consecutive order and flush with the ground. Both plates were covered with the same walkway surface material, so that targeting was not an issue. During one session, subjects walked until a minimum number of (usually) ten valid recordings were available. These recordings were defined as valid by the assessor when the participant walked naturally (e.g. with respect to targeting) and there was a clean foot strike on each force plate. Left and right foot contacts for each force plate were identified and set by visual inspection by the assessor during each recording. Patients were asked to walk at their self-selected walking speed. Healthy controls walked at three different walking speeds (mean and standard deviation, m/s): slow 0.98 (0.14), self-selected 1.27 (0.13), and fast 1.55 (0.15). In accordance with the internal rehabilitation center’s standards, patients walked either barefoot, with their orthopedic or normal shoes, and with or without orthopedic insoles. Healthy controls walked either barefoot or with their normal shoes. Prior to the gait analysis session, each participant underwent rigorous physical examination by a physician. The three analog GRF signals (vertical, anterior-posterior and medio-lateral force components) as well as the center of pressure (COP) were converted to digital signals using a sampling rate of 2000 Hz and a 12-bit analog-digital converter (DT3010, Data Translation Incorporation, Marlboro, MA, USA) with a signal input range of ±10 V. COP and GRF were recorded in the local force plate coordinate system (reaction-orientated). For easier usage the orientation of the medio-lateral and anterior-posterior signals for all data were uniformed, so that medial and anterior forces are always represented as positive values. Due to the center’s internal standards raw signals were only available down-sampled to 250 Hz. To avoid noise and signal peaks at the beginning and end of the signals, a threshold of 25 N was applied to all force data and the COP was calculated afterwards. These data are referred to as unprocessed (raw) GRF signals. Additionally, we have generated processed “ready to use” data. For this purpose the COP was only calculated when the vertical force reached 80 N to avoid inaccuracies in COP calculation at small force values. Additionally, the medio-lateral COP coordinates were mean-centered and anterior-posterior coordinates zero-centered. This was in line with the internal standards of the rehabilitation center. The processed force signals were then filtered using a 2nd order low-pass butterworth filter with a cut-off frequency of 20 Hz to reduce noise and were time-normalized to 100% stance (i.e. 101 points). The choice of appropriate cut-off frequency ranges widely in the literature, 20 Hz seems as a good trade-off between reducing noise and attaining as much physiological frequency content as possible^19^. The interested reader may also refer to [ref. ^20^, p.49]. Amplitude values of the three force components were expressed as a multiple of body weight (*BW*) by dividing the force by the product of body mass times acceleration due to gravity (g). Amplitude and time normalization are both necessary operations to reduce effects of covariates (such as anthropometry) on the signals and to reduce temporal differences which make comparisons of different steps difficult, e.g.^21,22^. Note that the processed and amplitude normalized data show small variations at the first and last frame of each signal. This might affect machine learning outcomes and therefore needs to be recognized. Sessions with less than three bi-lateral trials per participant were not included in the dataset. Additionally, we have used an algorithm proposed by Sangeux and Polak to eliminate any outliers before they were included in the GaitRec dataset^23^. This algorithm is based on the notion of depth, where the deepest signal is the equivalent to the median for univariate data and is sensitive to both shape and position of the signals. As suggested by Sangeux and Polak we have used a score of three to run their algorithm. All processing steps were performed in Matlab 2019a (The MathWorks Inc., Natick, MA, USA).

### Dataset & annotation

The presented dataset comprises completely anonymized GRF measurements from 2085 patients with different musculoskeletal impairments (“gait disorders”, GD) and data from 211 healthy controls (HC) including additional metadata such as age, sex, shod condition, walking speed condition, etc. For details see Table 1. Note that there is a considerable large gender imbalance in all GD classes. Healthy controls were recruited in the geographical region around the clinic’s by public posting and considered eligible if they were free of pain and complaints at the lower extremity and spine and did not have any orthotics or orthopedic insoles. Exclusion criteria were any history of surgery or trauma at the spine or lower extremities. This was assessed by an experienced therapist. A typical stay of a patient at the rehabilitation center ranged from a few days to several weeks and depends on factors such as diagnosis, administered therapy/surgery, and progress in recovery. During that time a patient is usually administered once a week to the gait analysis. At the beginning of a patient’s stay, therapy outcomes are mutually defined between the therapist and the patient. After reaching these goals in whole or in part, patients are usually discharged. However, they can be readmitted if necessary. The present dataset contains the data gathered during the entire stay(s) of each patient and covers a patient’s entire rehabilitation progress. Different types of analyses can thus be performed on the data set: an *inter-participant analysis* based on the initial assessment (first measurement session), e.g. for gait pattern classification, an *intra-participant analysis*, e.g. for the assessment of rehabilitation progress, or combinations.

**Table 1:** Demographic overview of the dataset and the pre-defined classes.

Class	N	Age (yrs.) Mean (SD)	Body mass (kg) Mean (SD)	Sex (m/f)	Bi-lateral Trials
Healthy C.	211	34.7 (13.9)	73.9 (15.6)	104/107	7,755
Hip	450	42.6 (12.8)	82.4 (15.6)	373/77	12,748
Knee	625	41.6 (12.0)	84.3 (18.6)	426/199	19,873
Ankle	627	41.6 (11.4)	87.0 (18.0)	498/129	21,386
Calcaneus	382	43.5 (10.4)	84.0 (14.5)	339/43	13,970
**Total**	**2,295**	**41.5 (12.1)**	**83.6 (17.3)**	**1,740/555**	**75,732**

**Fig. 1:**
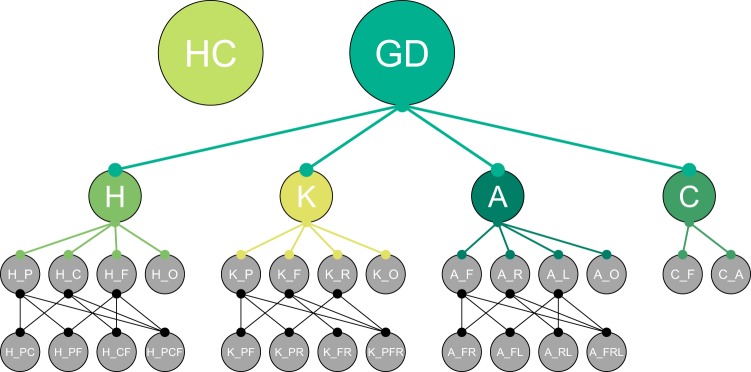
Class taxonomy. The class structure and the dependencies between the classes of the GaitRec dataset: Healthy Controls (HC), Gait Disorders (GD), Hip (H), Knee (K), Ankle (A), and Calcaneus (C). Details of the subclasses are described in Section Dataset & Annotation.

Regarding annotation, the dataset was manually labeled by a well-experienced physical therapist (with more than a decade of clinical experience) based on the available medical diagnosis of each patient. The annotation labels are formed by two strings concatenated with an underscore “*X_xxx*”, where “*X*” denotes the general anatomical joint level at which the orthopedic impairment was located, i.e. at the hip “*H*”, knee “*K*”, ankle “*A*”, or calcaneus “*C*”. The second string (“*xxx*”) gives a more detailed localization and is joint dependent, see the following paragraphs for details. An overview of the class structure is shown in Fig. 1.

- **Hip class (*H_xxx*):** The most common injuries present in the hip class are fractures of the pelvis and thigh as well as luxation of the hip joint, coxarthrosis, and total hip replacement. The second string “*xxx*” refers to the following specific anatomical regions: pelvis (H_P), coxa (H_C), the femur (H_F), and their combinations when two or more anatomical areas are affected (*H_PC, H_PF, H_CF, H_PCF*), as well as one class for other diagnoses (*H_O*).
- **Knee class (*K_xxx*):** The knee class comprises patients after patella, femur or tibia fractures, ruptures of the cruciate or collateral ligaments or the meniscus, and total knee replacements. The second string “*xxx*” refers to the following specific anatomical regions or diagnosis: patella (K_P), a fracture near the knee joint of the femur or the tibia (K_F), rupture of ligaments or the menisci (K_R), and their combinations (*K_PF, K_PR, K_FR, K_PFR*, as well as one class for other diagnoses (*K_O*).
- **Ankle class (*A_xxx*):** The ankle class includes patients after fractures of the malleoli, talus, tibia, or lower leg, and ruptures of ligaments or the Achilles tendon. The second string “*xxx*” refers to the following specific anatomical regions or diagnosis: fracture of the tibia, fibula or talus near the ankle joint (A_F), rupture of ligaments or the Achilles tendon (A_R), lower leg shaft fracture (A_L), and their combinations (*A_FR, A_FL, A_RL, A_FRL*, as well as one class for other diagnoses (*A_O*).
- **Calcaneus class (*C_xxx*):** The calcaneus class comprises patients after calcaneus fractures or ankle fusion surgery. The second string “*xxx*” refers to the following specific anatomical regions or diagnosis: fracture (C_F) or arthrodesis (C_A).

The hierarchical multi-level categorization allows for grouping the data into a dataset with four GD classes (H ∪ K ∪ A ∪ C) and one healthy controls (HC) class, but also holds more details if needed. Figure 1 and Table 1 give a brief overview of the dataset. Although the metadata includes a structured labelling of musculoskeletal impairments for each subject, there is no information available about the history of similar or other types of musculoskeletal injuries for both, the patient and the healthy controls. This limiting factors needs to be recognized when using GaitRec.

## Data Records

All published data are fully anonymized. The data records are available online from figshare^24^. The dataset consists of twenty files holding the GRF data (see Table 2) and one file holding the metadata, including the annotations and additional subjects’ information, e.g. category label, sex, body mass, etc. All files are available as comma-separated value files (CSV). The twenty GRF data files are organized according to the following naming convention: “*GRF-type-processing-side*.csv”. The *type* denotes, whether the file holds the vertical (“F_V”), anterior-posterior (“F_AP”), medio-lateral (“F_ML”) or the anterior-posterior or medio-lateral COP (“COP_AP”, “COP_ML”) time-series. *Processing* denotes, if the files hold the unprocessed raw data (“RAW”) or the post-processed data (“PRO”). The *side* denotes, if the data are from the “left” or “right” body side. The common prefix for all files is “GRF-”. An example filename is thus: “GRF_F_V_RAW_left.csv”.

**Table 2:** Description of the data stored in the “GRF_*.csv” files. “*” for the associated file name is a placeholder for “right” and “left”.

Variables	Associated file	Format	Dimension	Unit	Description
Vertical GRF	GRF_F_V-RAW_*.csv	double	1 × n	Newton	Raw vertical ground reaction force
Anterior-posterior GRF	GRF_F_AP-RAW_*.csv	double	1 × n	Newton	Raw breaking and propulsive shear force
Medio-lateral GRF	GRF_F_ML_RAW_*.csv	double	1 × n	Newton	Raw medio-lateral shear force
COP anterior-posterior	GRF_COP_AP_RAW_*.csv	double	1 × n	Centimeter	Raw COP coordinate in walking direction
COP medio-lateral	GRF_COP_ML_RAW_*.csv	double	1 × n	Centimeter	Raw COP coordinate in medio-lateral direction
Vertical GRF	GRF-F_V_PRO_*.csv	double	1 × n	Multiple of body weight	Post-processed vertical ground reaction force
Anterior-posterior GRF	GRF_F_AP_PRO_*.csv	double	1 × n	Multiple of body weight	Post-processed breaking and propulsive shear force
Medio-lateral GRF	GRF-F_ML_PRO_*.csv	double	1 × n	Multiple of body weight	Post-processed medio-lateral shear force
COP anterior-posterior	GRF_COP_AP_PRO_*.csv	double	1 × n	% stance	Post-processed COP coordinate in walking direction
COP medio-lateral	GRF_COP_ML_PRO_*.csv	double	1 × n	% stance	Post-processed COP coordinate in medio-lateral direction

n is either the number of frames during one step across the force plate for the unprocessed data (“RAW”) or a time-normalized vector of 101 points for the post-processed (“PRO”) data. Note that the first three columns of each file hold the SUBJECT_ID, SESSION_ID, and TRIAL_ID.

Each of the “*GRF-type-processing-side*.csv” files is structured as a matrix with *N* rows × *M* columns. Each row holds the data of one subject and trial. The first column identifies each subject (“SUBJECT_ID”), the second column each recording session (“SESSION_ID”), and the third column each single trial within a recording session (“TRIAL_ID”). Note that due to the non-normalized nature of the data and the resulting different vector lengths in the “RAW” files, non-available numbers have been replaced by “NaN” to maintain a constant matrix-dimension.

The metadata file, which contains annotations and additional subject-related information is available in “GRF-metadata.csv”. It is structured as a matrix with *N* rows × *M* columns (see Table 3). Here, the first two columns hold the SUBJECT_ID and SESSION_ID, the other columns hold information such as class labels, sex, body mass, age, shod-condition, see Table 3 for details. Note that this information is available in all records. Missing values are identified as “NaN”. A particularly notable field is “AFFECTED_SIDE”, which indicates which leg is affected by a certain impairment (e.g. left knee) or if both sides are affected.

**Table 3:** Description of the information stored in the metadata file.

Categories/Variables	Format	Unit	Description
**Identifiers**			
SUBJECT_ID	integer	—	Unique identifier of a subject
SESSION_ID	integer	—	Unique identifier of a session
**Labels**			
CLASS_LABEL	string	—	Annotated class labels
CLASS_LABEL_DETAILED	string	—	Annotated class labels for subclasses
**Subject Metadata**			
SEX	binary	—	female = 0, male = 1
AGE	integer	years	Age at recording date
HEIGHT	integer	centimeter	Body height in centimeters
BODY_WEIGHT	double	$\frac{kg\,m}{{s}^{2}}$	Body weight in Newton
BODY_MASS	double	kg	Body mass
SHOE_SIZE	double	EU	Shoe size in the Continental European System
AFFECTED_SIDE	integer	—	left = 0, right = 1, both = 2
**Trial Metadata**			
SHOD_CONDITION	integer	—	barefoot & socks = 0, normal shoe = 1, orthopedic shoe = 2
ORTHOPEDIC_INSOLE	binary	—	without insole = 0, with insole = 1
SPEED	integer	—	slow = 1, self-selected = 2, fast = 3 walking speed
READMISSION	integer	—	indicates the number of re-admission = 0 … n
SESSION_TYPE	integer	—	initial measurement = 1, control measurement = 2, initial measurement after readmission = 3
SESSION_DATE	string	—	date of recording session in the format “DD-MM-YYYY”
**Train-Test Split Information**			
TRAIN	binary	—	is part (=1) or is not part (=0) of TRAIN
TRAIN_BALANCED	binary	—	is part (=1) or is not part (=0) of TRAIN_BALANCED
TEST	binary	—	is part (=1) or is not part (=0) of TEST

**Fig. 2:**
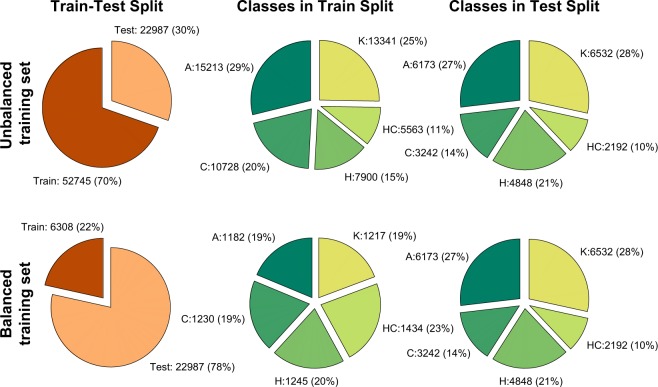
Dataset composition. Configuration of the balanced and unbalanced train/test splits of the GaitRec dataset. The pie-charts show the amount of trials populated (in total amount and percentage) within each class and split.

To foster comparability of classification results derived from the GaitRec dataset, we included a predefined randomized partitioning of the dataset into three subsets for training and testing. This information is stored in the metadata file. The GaitRec dataset is split into an unbalanced training set (TRAIN) and a test set (TEST). The first can be used for training and optimization of the machine learning models (e.g. by cross-validation) and the latter for the final evaluation. However, unbalanced classes might negatively affect the optimization of machine learning models, therefore we have created a balanced subset of TRAIN, referred to as TRAIN_BALANCED. The TRAIN_BALANCED subset comprises only data from initial assessments (first measurement session), which at least hold five trials for each body side per session. This is also the reason why the balanced splits populated sightly different amounts of trials. The data allocation to the different subsets was always performed on a random basis. Details of the train/test split configuration are depicted in Fig. 2.

## Technical Validation

**Fig. 3:**
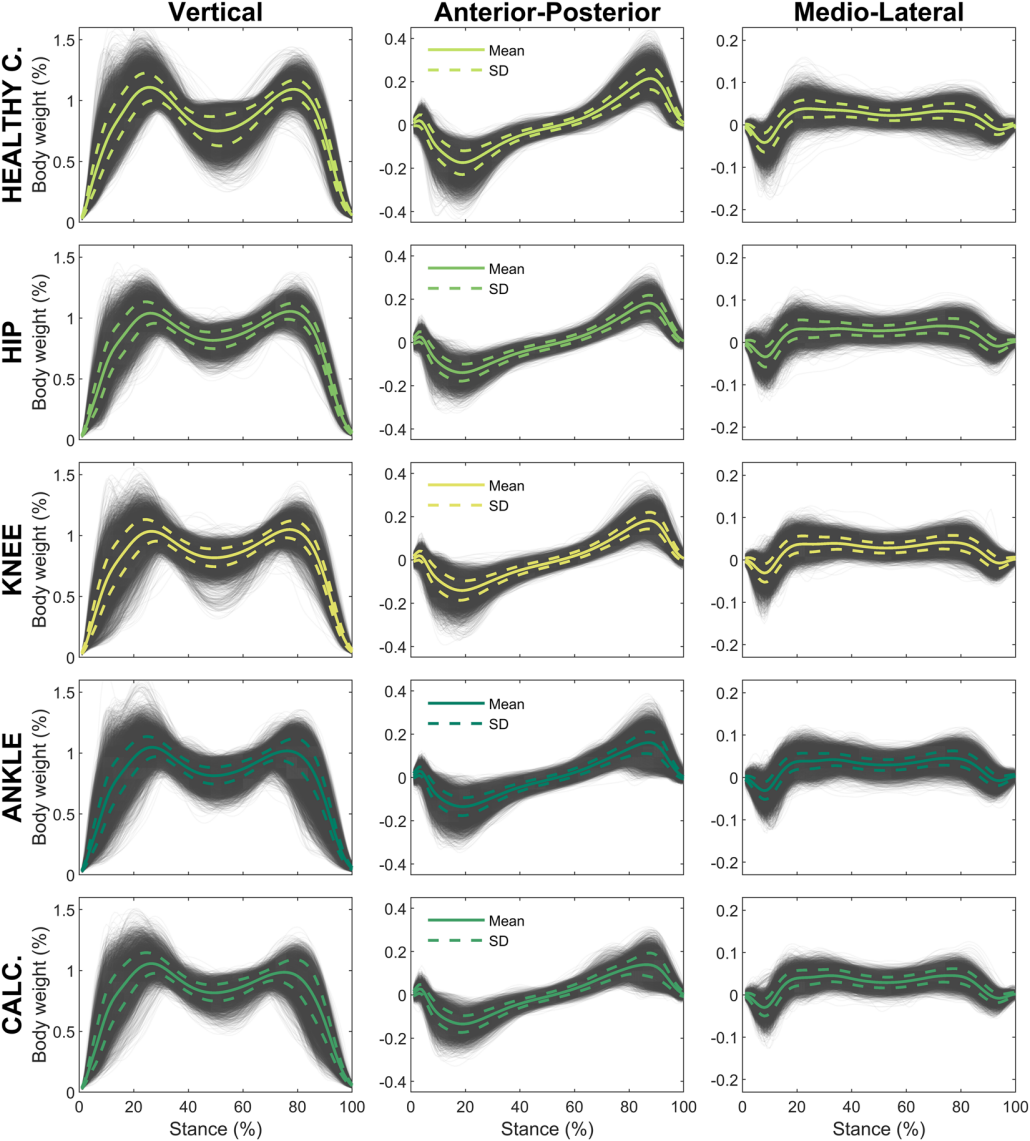
Data overview. Visualization of all body-weight normalized vertical, anterior-posterior, and medio-lateral GRF signals of the affected side available per subject and class. For healthy controls all available recordings are visualized. The plots also show the mean (solid line) and its one-fold standard deviation (dotted line). Note that for easier usage the orientation of the medio-lateral and anterior-posterior signals were uniformed, so that medial and anterior forces are always represented as positive values.

The provided data are available in raw format and post-processed with well-established de-noising and normalization procedures. This allows future researchers to either use the raw data and post-process them as desired (e.g., filtering, thresholding, normalization, etc.) or to employ the ready-to use post-processed data. The accuracy of the force plates was not specifically assessed during the data capturing period. However, the force plates and the measurement equipment has been checked and serviced regularly during clinical practice. To get a picture of the data integrity, the post-processed data are plotted in Fig. 3.

## Usage Notes

The data records are stored in *.csv files and can be easily imported into any desired software package for further data analysis. The dataset also contains two scripts which allow easy data import for Matlab (The MathWorks, Inc., Natick, Massachusetts, United States, 2019a) and Python (Python Software Foundation, 3.7). Benchmarks for automatically classifying the presented data based on the first annotation level into five classes, i.e. *H vs. K vs. A vs. C vs. HC*, can be found in our earlier work^13–15^. These works also provide a baseline approach that employs a signal representation based on Principal Component Analysis (PCA) combined with a Support Vector Machine (SVM) as a classifier for orientation and comparison. Note, however, that the presented dataset is an extended version of the dataset used in these studies and that results may thus slightly deviate from those of our previous studies. The studies further elaborate on the optimization of post-processing of GRF data for the purpose of gait classification.

Future work with the GaitRec dataset might focus on one of the research questions stated below. However, one should be aware that depending on the research question not all subsets of our dataset might be perfectly applicable due to their reduced sample size (i.e. for the balanced subsamples).

- Classifying healthy vs. pathological gait
- Build statistical models of normative walking
- Classify gait disorders
- Evaluation and prediction of therapy progress
- Gait data-record retrieval and similarity retrieval of trials
- Identification of subject-specific gait patterns
- Modeling dependencies between anthropometric/demographic data and the GRF signals

For the purpose of comparability of derived results from the GaitRec dataset, we highly recommend performing model optimization (e.g. by cross-validation) on the training set only and to keep the test set untouched until the final evaluation. However, it has to be noted that the train/test set split does not coincide exactly with the splits in our baseline experiments because both are larger now^13–15^.
